# Comparison of NOGA Endocardial Mapping and Cardiac Magnetic Resonance Imaging for Determining Infarct Size and Infarct Transmurality for Intramyocardial Injection Therapy Using Experimental Data

**DOI:** 10.1371/journal.pone.0113245

**Published:** 2014-11-19

**Authors:** Noemi Pavo, Andras Jakab, Maximilian Y. Emmert, Georg Strebinger, Petra Wolint, Matthias Zimmermann, Hendrik Jan Ankersmit, Simon P. Hoerstrup, Gerald Maurer, Mariann Gyöngyösi

**Affiliations:** 1 Department of Cardiology, Medical University of Vienna, Vienna, Austria; 2 Department of Biomedical Imaging and Image-guided Therapy, Medical University of Vienna, Vienna, Austria; 3 Swiss Centre for Regenerative Medicine, University of Zürich, Zürich, Switzerland; 4 Division of Surgical Research, University Hospital of Zürich, Zürich, Switzerland; 5 Clinic for Cardiovascular Surgery, University Hospital of Zürich, Zürich, Switzerland; 6 Department of Thoracic Surgery, Medical University of Vienna, Vienna, Austria; 7 Christian Doppler Laboratory for Cardiac and Thoracic Diagnosis and Regeneration, Vienna, Austria; University of Miami Miller School of Medicine, United States of America

## Abstract

**Objectives:**

We compared the accuracy of NOGA endocardial mapping for delineating transmural and non-transmural infarction to the results of cardiac magnetic resonance imaging (cMRI) with late gadolinium enhancement (LE) for guiding intramyocardial reparative substance delivery using data from experimental myocardial infarction studies.

**Methods:**

Sixty domestic pigs underwent diagnostic NOGA endocardial mapping and cMRI-LE 60 days after induction of closed-chest reperfused myocardial infarction. The infarct size was determined by LE of cMRI and by delineation of the infarct core on the unipolar voltage polar map. The sizes of the transmural and non-transmural infarctions were calculated from the cMRI transmurality map using signal intensity (SI) cut-offs of>75% and>25% and from NOGA bipolar maps using bipolar voltage cut-off values of <0.8 mV and <1.9 mV. Linear regression analysis and Bland-Altman plots were used to determine correlations and systematic differences between the two images. The overlapping ratios of the transmural and non-transmural infarcted areas were calculated.

**Results:**

Infarct size as determined by 2D NOGA unipolar voltage polar mapping correlated with the 3D cMRI-LE findings (r = 0.504, p<0.001) with a mean difference of 2.82% in the left ventricular (LV) surface between the two images. Polar maps of transmural cMRI and bipolar maps of NOGA showed significant association for determining of the extent of transmural infarction (r = 0.727, p<0.001, overlap ratio of 81.6±11.1%) and non-transmural infarction (r = 0.555, p<0.001, overlap ratio of 70.6±18.5%). NOGA overestimated the transmural scar size (6.81% of the LV surface) but slightly underestimated the size of the non-transmural infarction (−3.04% of the LV surface).

**Conclusions:**

By combining unipolar and bipolar voltage maps, NOGA endocardial mapping is useful for accurate delineation of the targeted zone for intramyocardial therapy and is comparable to cMRI-LE. This may be useful in patients with contraindications for cMRI who require targeted intramyocardial regenerative therapy.

## Introduction

The border zone of myocardial infarction (MI) represents myocardial areas with decreased viability and reduced wall motion capacity. Perfusion and transport of cell-death waste products, such as oxygen radicals and other metabolic substances, is impaired due to the close proximity to the non-perfused infarcted area, and this may account for the functional decline. These areas are targeted by cardiac regenerative therapies because regenerative cells delivered to these areas may survive and help restore cardiac function.

Compared to intracoronary or intravenous delivery, intramyocardial delivery of regenerative drugs, genes, or cells into the border zone of chronic myocardial ischemia results in higher retention of the applied substances, which may result in more effective therapy [1]–[4]. However, accurate real-time localization of this area for application of intramyocardial regenerative therapy remains a challenge. Cardiac magnetic resonance imaging (cMRI) with late gadolinium enhancement (LE) is the gold standard for assessing myocardial infarct size, infarct transmurality, and left ventricular (LV) function and for assessing the efficacy of cardiac therapies [5], [6]. Identification of subendocardial or non-transmural infarcted areas using cMRI-LE would be ideal for guiding targeted intramyocardial regenerative therapy. However, cMRI is an off-line imaging modality, and there is a delay between diagnostic imaging and application of the therapy. Further, cMRI is contraindicated for patients with cMRI-non-compatible pacemakers or implantable defibrillators.

Three-dimensional (3D) NOGA endocardial mapping and electromagnetic guided percutaneous intramyocardial therapy is the method that is currently used for real-time (on-table) assessment of myocardial viability and for delineation of the infarct and infarct border zone [7]–[10]. The accuracy and reproducibility of NOGA maps for evaluating myocardial viability have been established [11]–[15], and this 3D imaging method has been compared with other 3D imaging methods such as myocardial scintigraphy, positron emission tomography, and cMRI [16]–[20]. Furthermore, histology, echocardiography, and other methods have confirmed that NOGA mapping can be used to correctly assess the size and severity of myocardial necrosis [21]. In order to assess the accuracy of the point-to-point sampling method of NOGA mapping, several research groups have developed fusion software for constructing hybrid images of cMRI and NOGA CARTO mapping [22]–[29]. These reports on a limited number of patients confirmed that there is good correlation between the two 3D images regarding the location and size of the infarction. However, the reports noted that NOGA mapping does not show good correlation with cMRI-LE in terms of the delineation of non-transmural areas. Moreover, the aim of these multimodality images was to find a focus for arrhythmogen substrates for ablation therapy to treat reentry tachycardias using unipolar and bipolar voltage electrocardiograms.

Here we focused on the accuracy of NOGA mapping to delineate transmural and non-transmural infarction by comparing it with cMRI-LE imaging. We investigated whether NOGA mapping is suitable for guiding intramyocardial drug or cell delivery using data from experimental myocardial infarction studies. We chose to use an animal model of closed chest reperfused MI. This is very similar to human primary percutaneous coronary intervention in acute MI, which simulates post-infarction left ventricular dysfunction.

We hypothesized that real-time, on-table 3D endocardial mapping using the NOGA system can accurately delineate the zone of decreased viability and non-transmural scars that is the target area for percutaneous intramyocardial therapy. Here we show that there is a significant correlation between the two images in terms of infarct size and the sizes of the transmural and non-transmural infarction, with high degree of overlap between endocardial mapping and cMRI-derived infarcted areas.

## Materials and Methods

### Experimental chronic left ventricular dysfunction post MI

Closed-chest reperfused acute MI was induced in 60 female farm pigs by percutaneous occlusion of the mid left anterior descending coronary artery. Sixty days later, the pigs underwent cMRI-LE images to confirm chronic myocardial infarction. Additional NOGA endocardial mapping was then performed 2±1 days later, just before the animals were euthanized, aiming to search for correlations between the two images.

Briefly, all pigs were sedated with ketamine hydrochloride (12 mg/kg), xylazine (1 mg/kg), and atropine (0.04 mg/kg) intramuscularly. The pigs were intubated intratracheally and anesthetized with isoflurane (1.5–2.5 vol%), O_2_ (1.6–1.8 vol%), and N_2_O (0.5 vol%). O_2_ saturation, blood pressure, and electrocardiography were monitored continuously. After an arteriotomy of the right femoral artery, a 6F introducer sheath (Terumo Medical Corporation, Somerset, NJ, USA) was inserted. Heparin (200 IU/kg) was administered followed by selective angiography of the left coronary artery tree. A balloon catheter (3.0 mm in diameter, 9 mm long; Maverick, Boston Scientific Corp, Natick, MA, USA) was advanced into the left anterior descending coronary artery (LAD). After the origin of the second major diagonal branch, the mid LAD was occluded by inflation of the balloon at 5 atmospheres for 90 minutes, followed by deflation of the balloon to allow opening of the infarct-related artery and reperfusion of the ischemia-affected myocardium. The pigs were then allowed to recover from the anesthesia.

This study was carried out in strict accordance with the recommendations in the Guide for the Care and Use of Laboratory Animals of the National Institutes of Health. The protocol was approved by the Experimental Animal Care and Use Committee at the Faculty of Animal Science of the University of Kaposvar (Hungary) (Permit Numbers: 246/002/SOM2006/11/11 and 246/002/SOM2006/08/11). All procedures were performed under general anesthesia, and all efforts were made to minimize the suffering of the animals.

### NOGA electroanatomical mapping of the LV

The electromechanical maps of the pigs were obtained 62±1 days after induction of acute MI. The principles and procedures of NOGA electroanatomical mapping have been described previously [7]–[10]. Briefly, the external reference patch, which contains a tip sensor, was taped onto the experimental animal's back below its heart. A 7 F introducer sheath was introduced into the left femoral artery, followed by administration of 5000 IU of unfractionated heparin. To begin the navigation process, a fully deflected NogaStar (Johnson & Johnson, Diamond Bar, CA) mapping catheter was introduced through the femoral sheath and was advanced inside the aorta under fluoroscopic guidance. Before entering the LV chamber, the mapping catheter was bent and then introduced through the aortic valve. As soon as the tail of the catheter was inside the LV, the catheter tip was straightened and orientated towards the apex. The first few endocardial points in different regions of the LV (apical point, outflow tract, and lateral and posterior points) were sampled to initiate a three-dimensional silhouette of the heart, which was then updated in real time with every new mapping point which facilitated further electromagnetic navigation with limited use of fluoroscopy.

At least four points were acquired in each of twelve myocardial segments. During the mapping process, simultaneous unipolar and bipolar electrocardiograms were recorded as well as the 3D location and orientation of the catheter tip of several sites within the LV cavity. The electrophysiological and mechanical data were integrated to create a color-coded 3D reconstruction of the LV chamber to facilitate assessment of the regional viability. The stability of the catheter-to-wall contact was evaluated at every location in real time, and only stable location points were accepted to create the 3D maps [9], [10]. When constructing the NOGA maps, care was taken to correctly define the apex and the heart axis.

Using the movement of the catheter tip in the LV cavity, the end-diastolic, end-systolic volumes and global ejection fraction were calculated automatically. The average heart rate was calculated during the electroanatomical mapping procedure.

### Evaluation of the NOGA maps

**Figure 1:**
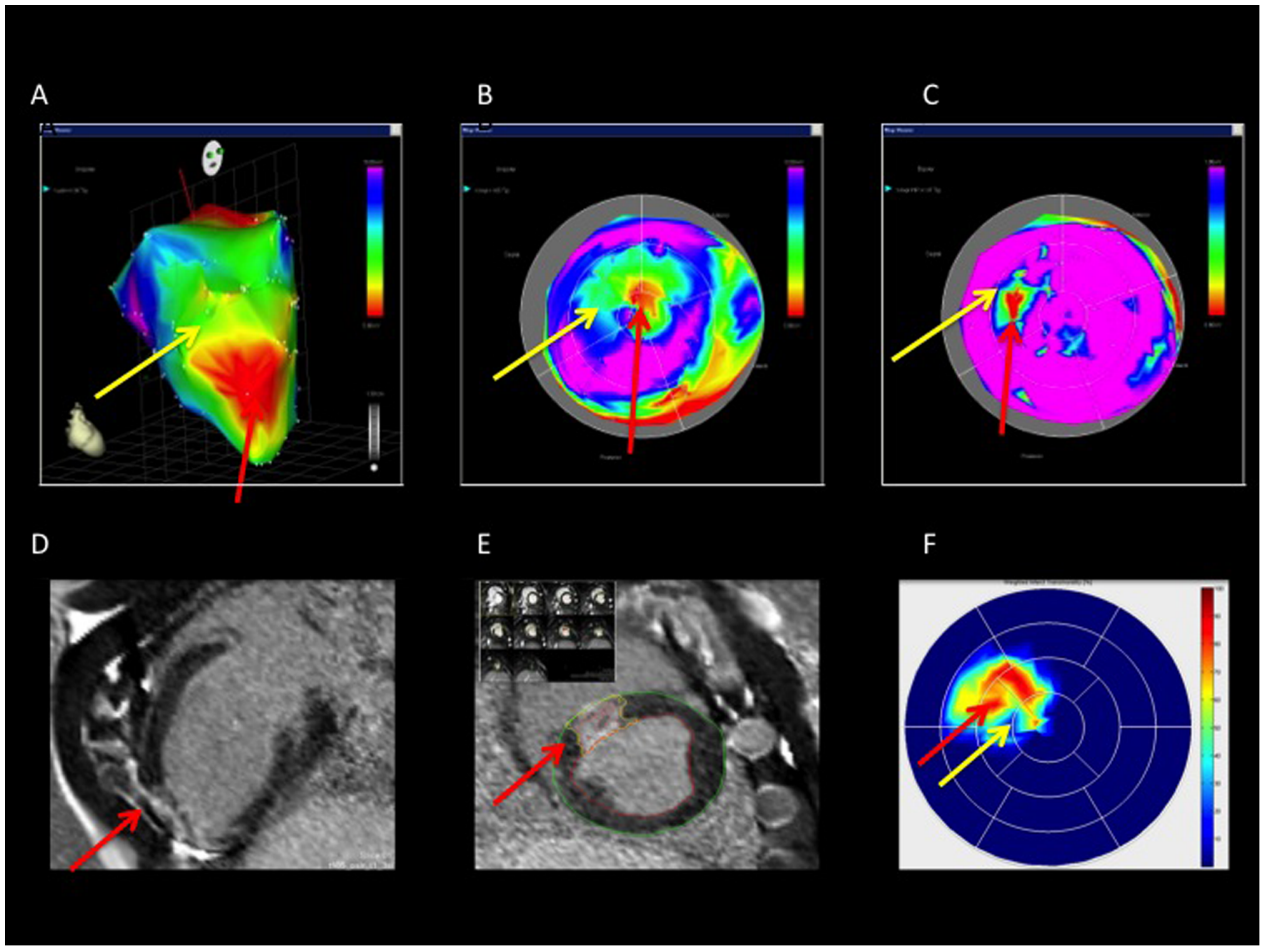
NOGA endocardial mapping and cardiac magnetic resonance imaging (cMRI) of a pig with chronic myocardial ischemia. A. 3D NOGA mapping showing anterior, anteroseptal, and apical myocardial infarction. Red indicates the infarct core (red arrow), the surrounding green-yellow area shows the border zone of infarction (yellow arrow). B. The corresponding unipolar voltage polar map. The color-coding is the same as in A. C. The bipolar voltage polar map of the same pig. Red indicates the transmural infarction (red arrow); yellow-green indicates the non-transmural infarction (yellow arrow), and blue-pink indicates normal myocardium. D. cMRI with late enhancement reveals the myocardial scar (red arrow). Long axis image. E. cMRI late enhancement short axis images with myocardial infarction (red arrow). Quantitative size of infarction was assessed by dividing of the myocardium to 8 slices from heart basis to apex (left upper corner). F. A cMRI transmurality polar map shows the transmural infarction (red arrow) with a surrounding area of non-transmural infarction (yellow arrow).

**Figure 2:**
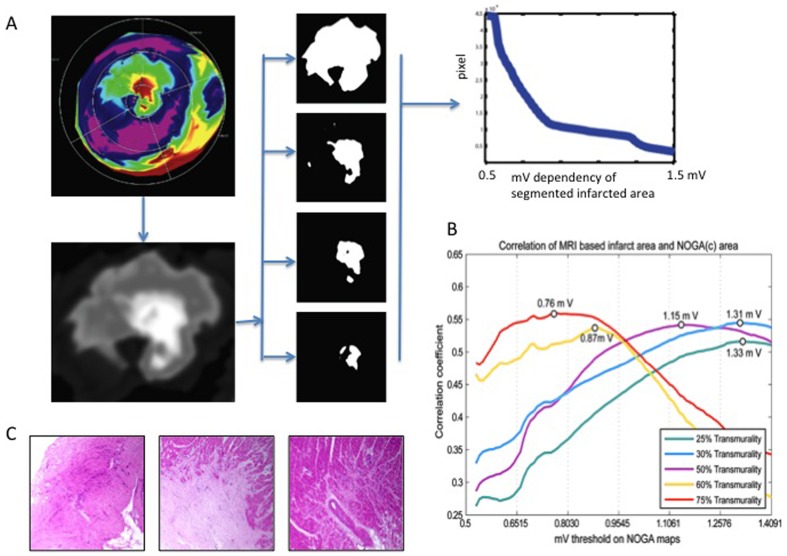
Magnet resonance imaging- (MRI-) derived NOGA bipolar threshold values for infarct transmurality. A. Iterative thresholding of voltage maps (left panel). The original NOGA maps were cropped to infarct areas, converted to a mV scale, then iteratively thresholded using 100 steps from 0.5 to 1.5 mV. The middle panel shows examples of the iterative thresholding process. The right panel shows the association between the area of infarction and the applied mV threshold. B. Voltage-threshold dependence of the correlation between NOGA maps and areas on an MRI. The maximum correlation coefficient is shown for each line plot. C. Histological correlations of transmural (left) and non-transmural (middle) infarctions and normal heart tissue (right). Samples were taken from areas with bipolar voltage values <0.8 mV, between 0.8–1.9 mV, and>1.9 mV with corresponding cMRI-LE transmurality values of>75%, 50%, and <25%, respectively. Magnification: 2x.

ImageJ for Windows (U.S. National Institutes of Health [NIH], Bethesda, Maryland, USA) was used to determine the relative size of the area of interest. The 3D unipolar (UPV) and bipolar (BiPV) voltages of each measuring point were displayed as two-dimensional polar maps (bull's eye format). The same discriminatory threshold values were used for all NOGA maps: <5 mV for non-viable infarcted area, 5–15 mV for infarct border zone and>15 mV for normal viability in unipolar maps and <0.8 mV for transmural, 0.8–1.9 mV for non-transmural infarction and>1.9 mV for normal myocardium in bipolar maps. These cut-off values are represented by the color-coding of the NOGA images and facilitate the identification of areas with reduced UPVs and BiPVs (Table 1, Fig. 1). As a next step, in order to validate the discriminatory threshold values for bipolar NOGA maps, we determined the correlation between MRI-based infarct area measurements and different discriminatory bipolar cut-off values. Specifically, we exported the raw images of the NOGA transmurality data (i.e. the bipolar recordings) into the image processing pipeline of Matlab R2010 for Windows, converted the image RGB (red-green-blue) color values into voltage values, and cropped the image to the cMRI-LE infarct area. Using 100 threshold steps (from 0.5 to 1.5 mV), we iteratively exported the NOGA-based transmurality areas to the cMRI transmurality maps and determined the correlation coefficient between the two images using different thresholds (25, 30, 50, 60, and 75% transmurality) (Fig. 2A).

**Table 1:** NOGA endocardial unipolar and bipolar map-derived cut-off values.

Cut-off value	Color on the NOGA map	Definition
Unipolar voltage map		
>15 mV	Blue, violet	Normal tissue
5–15 mV	Yellow, green	Border zone of infarction
<5 mV	Red	Area of myocardial infarction
Bipolar voltage map		
>1.9 mV	Blue, violet	Normal tissue
0.8–1.9 mV	Yellow, green	Non-transmural infarction
<0.8 mV	Red	Transmural infarction

**Figure 3:**
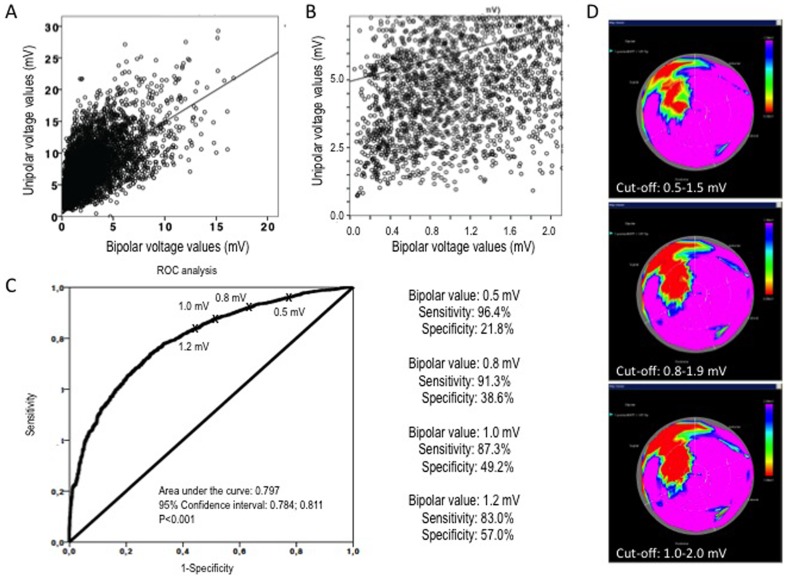
Determination of the bipolar voltage (BiPV) cut-off value for calculating the transmural and non-transmural infarct size using unipolar voltage values. A. Linear correlation between unipolar and bipolar voltage values. B. Part of the correlation showing the non-viable range defined as unipolar voltage values <5 mV. C. Receiver operator characteristics curve for determination of different bipolar voltage cut-off values. D. Display of bipolar maps using different cut-offs.

In order to determine the BiPV cut-off that provides the best fit to the size of transmural and non-transmural cMRI-LE areas, we also tested 0.5–1.5 mV and 1.0–2.0 mV BiPV cut-offs (Fig. 3). The fibrous ring and cardiac valves, represented as red zones at the outer edge of the bull's eye NOGA maps, were excluded from analysis as they contained low UPVs and BiPVs that were indicative of non-contractile collagen structures [9], [10].

**Figure 4:**
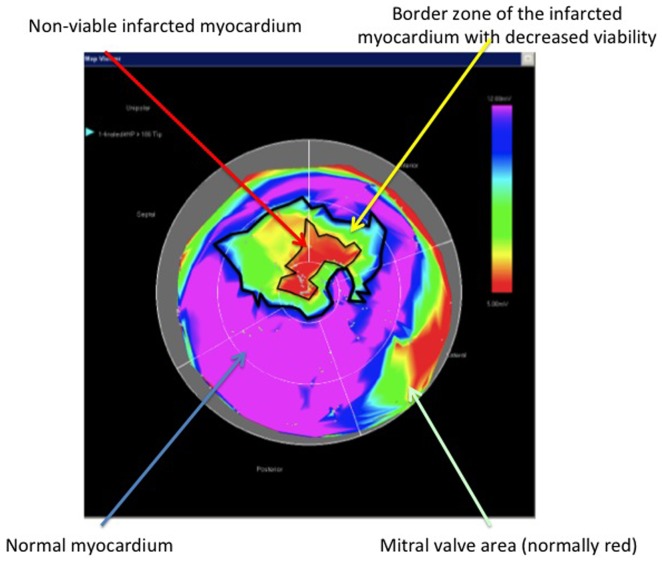
Schematic illustration of the planimetric calculation of the size of the infarct core and the size of the border zone of the infarction with decreased viability in the NOGA endocardial mapping. Red indicates the infarct core, and green-yellow indicates the surrounding area that has decreased viability.

**Figure 5:**
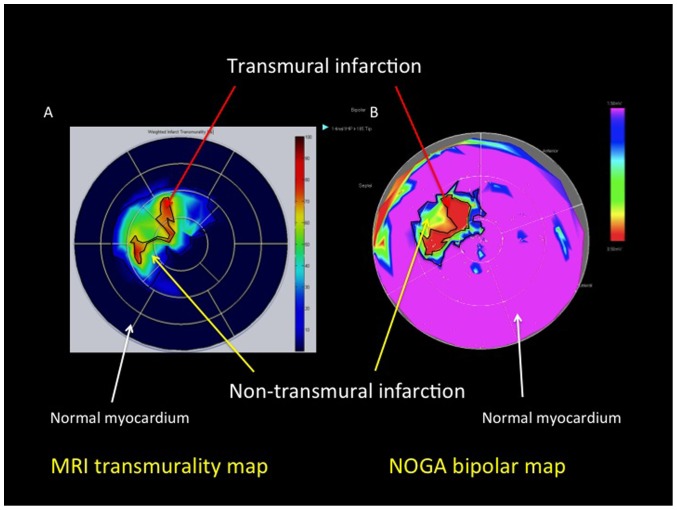
Schematic illustration of the planimetric calculation of the transmural and non-transmural infarct sizes as shown in polar maps from cardiac magnetic resonance imaging (cMRI) and NOGA endocardial mapping. A. Planimetric calculations of the sizes of the transmural and non-transmural infarction by cMRI. B. Planimetric calculations of the sizes of the transmural and non-transmural infarction in a NOGA bipolar voltage map.

The relative size of the infarct core, border areas, and normal myocardium was determined from the color-coded unipolar voltage polar map, and the sizes of the transmural and non-transmural infarction were delineated on the bipolar maps (Figs. 4 and 5). The demarcation and measurement of these areas on the NOGA maps was carried out by a researcher who was blinded to the results of the cMRI-LE.

### cMRI-LE acquisitions

Cardiac magnetic resonance (cMRI) images were acquired on a 1.5 T Siemens Avanto Syngo B17 clinical scanner (Erlangen, Germany) with a phased-array coil and a vector ECG system. Functional (dynamic) images were acquired using a retrospective ECG-gated (HR: 80–100 beats/minute), steady-state free precession (SSFP - TRUFISP sequence) technique in short-axis and long-axis sections of the heart using 1.2-ms echo time (TE), 40-ms repetition time (TR), 25 phases, 50° flip angle, 360-mm field-of-view, 8-mm slice thickness, and a 256×256 image matrix. For quantitative measurement of infarct extent and transmurality, delayed enhancement diastolic phase short-axis images were obtained after injection of 0.05 mmol/kg of contrast medium using an inversion recovery prepared gradient-echo sequence. LE images were obtained 10 minutes after gadolinium contrast agent injection.

### Analysis and visualization of cMRI images

In all cases, care was taken to include the entire heart volume from the apex to the level of the great vessels, and the entire left ventricle was included in the MRI analysis. Analysis was consistently restricted to slices between the apex and the basis on which the left ventricle myocardium is seen in 360 degrees. Image analysis was performed using Segment for Windows software (version 1.9; Medviso AB, Lund, Sweden) [30]. The extent and transmurality of the infarct was semi-automatically quantified on the 10-minute LE images using the “2SD” approach. During this procedure, hyperintense image pixels were tagged as infarcted if their signal intensity (SI) was greater than the mean value plus 2 standard deviations of the normal-appearing LV myocardium. Signal intensity was derived from the absolute value of the voxel intensity data, which were stored in the DICOM files from the cMRI-LE acquisitions.

The 3D infarct volume was determined both in absolute units and as a percentage of the total LV myocardium volume (Fig. 1). The entire LV was imaged by both NOGA and cMRI-LE in order to obtain an accurate volume measurement. The non-contractile mitral valve areas, which are clearly visible in both images, were excluded from the quantitative analysis of infarct size and transmurality.

Polar plots were derived from the infarct segmentations using segmental transmurality data. The corresponding bull's eye maps were processed to achieve the same apex, heart axis, and orientation as for NOGA maps.

For these visualizations, viability data from 8 apico-basal slices and 32 wall sectors per slice were interpolated and smoothed. The linear borders of 17 segments were overlaid on the images graphically to help interpret the images using “classical” segmental nomenclature, while the interpolated data originated from 32 sections per slice. The endo- and epicardial borders were segmented, and a segmental model was fitted on the left ventricle model (32 sections per slice). Using the standard transmurality analysis feature of the Segment software (Medviso Inc., Lund, Sweden), the midline was calculated for each section of the LV myocardium. The transmurality of the infarct is given as the radial projection of the segmented scar volume on the midline, summed for each section.

By applying two-dimensional planimetry to the polar plots, we performed two distinct measurements of the transmurality of the infarct. The extents of the transmural or non-transmural infarct and normal areas were defined as the percentage of sectors in which the SI was>75% (infarct core), 51–75% (border zone of infarction), 25–50% (non-transmural infarction), or <25% (normal) [22]. Alternatively, SI>60% was defined as transmural infarct, 31–60% as non-transmural infarct, and <30% as a normal area [21]. To calculate the areas, we used the raw transmurality data from 32 sections per slice, as noted above. All MRI area measurements are reported as the percentage of segments in the threshold range divided by the total number of segments.

Dynamic MR images were analyzed for myocardial wall movement and functional parameters. We carried out semi-automatic segmentation of the LV endocardial and epicardial borders while the end-diastolic volume (EDV), end-systolic volume (ESV), global LV ejection fraction (EF), and cardiac output (CO) were calculated automatically on short-axis images.

### Overlap ratio

**Figure 6:**
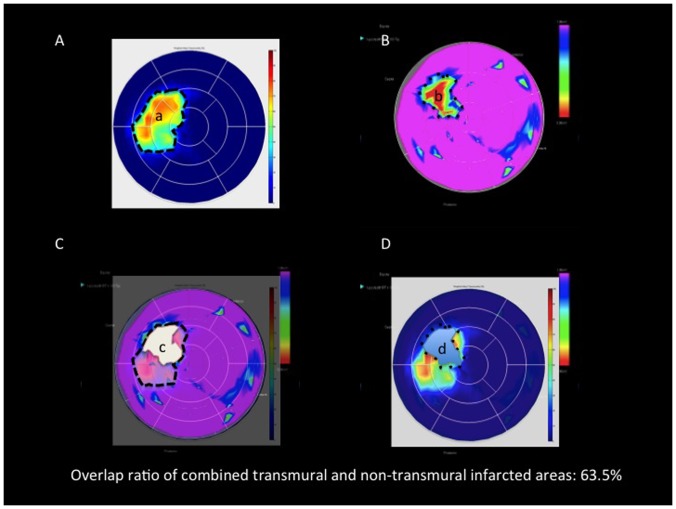
Calculation of the overlapping ratio of the transmural plus the non-transmural infarction by cardiac magnetic resonance imaging (cMRI) and NOGA endocardial mapping. A. cMRI transmurality polar map. B. NOGA bipolar polar map. C and D. Overlap of the cMRI and NOGA polar maps. a: The size of the transmural and non-transmural infarction by cMRI; b: the size of transmural and non-transmural infarction by NOGA bipolar mapping; c: overlap of the NOGA bipolar infarct shape on a cMRI polar map; d: overlap of the cMRI infarct shape on a NOGA bipolar map. The overlapping ratio was calculated as follows: (c+d)/(a+b).

The overlap ratio between the cMRI and NOGA bipolar transmurality maps was calculated as follows [28] (Fig. 6):

(NOGA bipolar map overlap area + cMRI transmural map overlap area)/(NOGA bipolar low voltage area + cMRI transmurality area)

For the overlap ratio of the transmural, non-transmural and the combined transmural and non-transmural (not normal) areas the following cut-off values were used:

Transmurality overlap ratio: between a NOGA bipolar map area with BipV <0.8 mV and a cMRI transmurality area with SI>75%;
Non-transmural areas overlap ratio: between a NOGA bipolar map area with BipV 0.8-1.9 mV and a cMRI transmurality area with SI 25–75%;
Combined transmural and non-transmural (not normal) area overlap ratio: between a NOGA bipolar map area with BipV <1.9 mV and a cMRI transmurality area with SI>25% (Figs. 4 and 5).

Overlap>60% was considered good accuracy [28], and 50–60% overlap was considered moderate accuracy (arbitrary value).

### Histology

Myocardial samples were collected from transmural and non-transmural infarctions and from normal heart tissue. These were areas with bipolar voltage values <0.8 mV, from 0.8–1.9 mV, and>1.9 mV with corresponding cMRI-LE transmurality values of>75%, 50%, and <25%, respectively. The samples were stored in 4.5% buffered formalin for at least 24 h and embedded in paraffin. Sections were cut into 4- to 6-µM thick slices and stained with hematoxylin-eosin.

### Statistical analysis

Continuous variables are presented as mean values ± standard deviation. The correlation between NOGA- and cMRI-derived parameters (infarct size, transmural and non-transmural infarct size) was calculated by linear regression analysis using Pearson Product Moment Correlation and Bland-Altman plots. Linear regression and Bland-Altman analysis was performed using Matlab R2010 software for Windows. During the iterative fitting of the linear function, a “least absolute residual” robust regression approach was used [31]. For interpretation of the correlations between 2 images the standardized nomenclature was used: if the correlation coefficient is greater than 0.5 is large, 0.5–0.3 is moderate, and 0.3–0.1 is a small correlation [32].

In order to choose the correct BiPV cut-off values for determining the transmural and non-transmural scars, all UPVs were also correlated with the corresponding BiPVs using linear regression analysis. Using a 5 mV UPV value as the cut-off for viability/non-viability, receiver operator curve (ROC) analysis was performed, and the sensitivity, specificity of different BiPV values, and the area under the curve, with 95% confidence intervals, were calculated.

For all statistical analyses, a p-value <0.05 was considered statistically significant.

## Results

There were no complications during the NOGA and cMRI-LE procedures that required additional medication or cardiopulmonary resuscitation.

### Correlation of NOGA threshold values with MRI-based transmurality

Depending on the threshold values applied to the NOGA bipolar voltage maps, the correlations between the different cut-offs of the two images were r = 0.27–0.57. The 75% MRI transmurality areas showed the best correlation with the NOGA-based areas using a cut-off of 0.76 mV, while the 60% transmurality areas correlated best with NOGA maps using a 0.87 mV threshold. The validation experiment resulted in a cut-off value of 1.3 mV for 25% transmurality. In order to exclude non-transmural ischemia, we used the usual value of 1.9 mV for normal, non-infarcted areas (Figure 2B). Notably, these cut-offs correlated well with the histological findings (Fig. 2C), confirming the proprietary diagnosis of transmural and non-transmural areas.

### cMRI and NOGA mapping results

The cMRI-LE and NOGA endocardial mapping results are summarized in Tables 2 and 3. NOGA mapping and cMRI-LE showed similar results for LV volumes and global EF. Heart rates were slightly higher during the NOGA procedure, probably due to the longer procedure time.

**Table 2:** Endocardial NOGA mapping results.

NOGA electromechanical mapping	Value (mean ±SD) (n = 60)
End-diastolic volume	108±12 mL
End-systolic volume	67.1±9.6 mL
Stroke volume	41.3±11.6 mL
Left ventricular ejection fraction	38.7±7.6%
Heart rate	110±7 bpm
Relative size of the infarct core (area of unipolar voltage <5 mV)	19.5±8.1%
Size of the area of border zone of the infarction (area of unipolar voltage 5–10 mV)	20.4±9.7%
Size of the transmural infarction (area of bipolar voltage <0.8 mV)	14.1±6.2%
Size of the non-transmural infarction (area of bipolar voltage 0.8–1.9 mV)	11.6±5.6%

**Table 3:** Results of cardiac magnetic resonance (cMRI) with late enhancement.

cMRI	Value (mean ±SD) (n = 60)
End-diastolic volume	106±22 mL
End-systolic volume	67.8±22 mL
Stroke volume	38.2±8.4 mL
Left ventricular ejection fraction	37.6±8.9%
Heart rate	102±11 bpm
Cardiac output	4.2±1.1 L/min
Cardiac index	3.7±0.9 L/min/m^2^
Left ventricular myocardial mass	108±15 g
Left ventricular myocardial volume	103±14 mL
Relative infarct size of left ventricular myocardial mass	16.9±6.3%
Volume of myocardial infarction	15.7±6.5 mL
0%–30%–60%–100% SI for transmurality	
Size of transmural infarction if SI>60%	10.4±5.1%
Size of non-transmural infarction if SI = 31–60%	8.4±3.9%
Size of non-infarcted tissue if SI<30%	81.2±6.7%
0%–25%–50%–75%–100% SI for transmurality	
Size of transmural infarction if SI>75%	6.4±5.6%
Size of border zone of transmural infarction if SI = 51–75%	7.5±4.3%
Size of non-transmural infarction if SI = 25–50%	6.7±2.9%
Size of non-infarcted tissue if SI<25%	79.4±7.6%

SI: signal intensity.

The NOGA endocardial maps contained a mean of 202±31 (range: 152–295) mapping points per animal, with a mapping point distribution of 16.8±5.5 points per segment of the 12-segment view. The mapping points were connected automatically if the distance between points was less than 15 mm. The mean time for the NOGA procedure was 35±9 min.

The BiPV values correlated significantly with the UPV values (Fig. 3). ROC analysis showed that a BiPV value of 0.8 mV as the non-transmural cut-off value had a sensitivity>90% and low but acceptable specificity. Display of the bipolar maps with different cut-offs revealed small differences between the images, with a transmural infarct size of 12.5±8.7% using a 0.5-mV cut-off value, 14.1±6.2% using a 0.8-mV cut-off value, and 16.8±5.3% using a 1-mV BiPV cut-off value, with no statistically significant differences between the 3 area measurements. Based on the best overlap ratio between the two images, a BiPV cut-off of 0.8–1.9 mV was used for further analysis.

The unipolar voltage map-derived scar size (UPV<5 mV) showed a moderate association with the transmural scar size as determined by bipolar maps (BiPV<0.8 mV) (r = 0.385, p = 0.002). There was better concordance between the border zone of infarction (UPV 5–15 mV) and non-transmural scars (BiPV 0.8–1.9 mV) (r = 0.457, p<0.001). The total size of the ischemic area showed good correlation between the two maps (r = 0.575, p<0.001).

### Correlation of infarct size as determined by cMRI-LE and NOGA mapping

**Figure 7:**
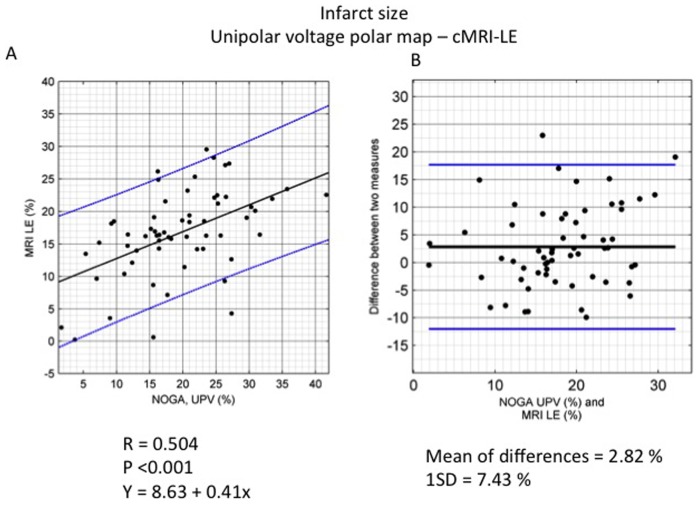
Correlation between NOGA endocardial mapping and cardiac magnetic resonance imaging (cMRI) and statistical analysis of the determination of infarct size. A. Regression equation between the size of the infarct core as determined on a unipolar voltage map (UPV) and as determined using late enhancement cMRI. B. Bland-Altman plot of the size of the infarct core as determined on a unipolar voltage map and the size as determined using late enhancement cMRI. Mean (black line) ±2SD (blue line).

There was a significant correlation (r = 0.504, p<0.001) between the infarct size as determined using the NOGA unipolar voltage polar map and the size determined by cMRI-LE. The Bland-Altman plot showed that NOGA mapping resulted in a systematically higher infarct size compared to cMRI-LE, with a mean difference between the two images of 2.82±7.43% in the LV surface (Fig. 7).

### Correlation of transmural infarct size as determined by cMRI and NOGA mapping

Using SI>75% and NOGA BipV <0.8 mV, the correlation between the two images was r = 0.727 (p<0.001) for the size of the transmural infarction. NOGA bipolar mapping resulted in a larger transmural infarction size than did cMRI-LE, with a mean difference of 6.81±4.39%) in the LV surface.

Using cut-offs for infarct transmurality of SI>60% and NOGA BipV<0.8 mV, the correlation was slightly lower (r = 0.576, p<0.001), with a mean difference between the two images of 2.14%±5.62% of LV surface.

### Correlation between cMRI and NOGA mapping regarding non-transmural infarct size

**Figure 8:**
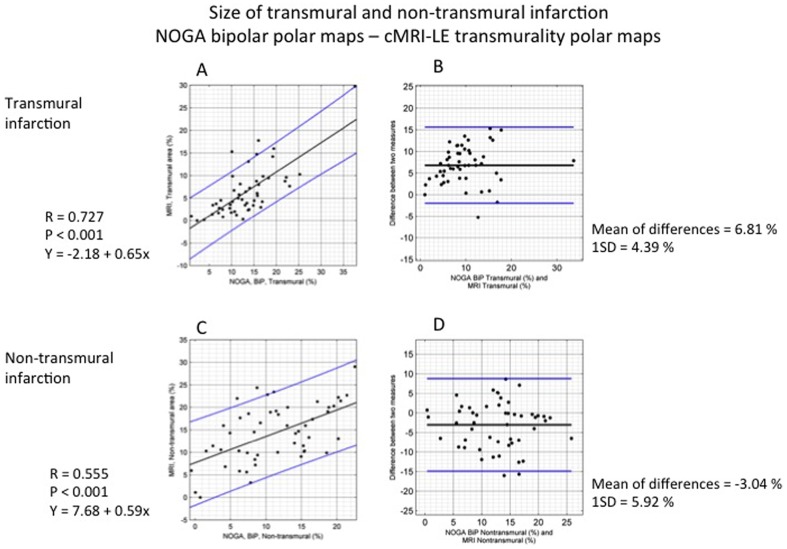
Correlation between NOGA endocardial mapping and cardiac magnetic resonance imaging (cMRI) and statistical analysis of the determinations of transmural and non-transmural infarction. A. Regression equation between the size of the transmural infarction as determined on a bipolar voltage map and the size as determined using cMRI. B. Bland-Altman plot of the size of the transmural infarction as determined on a bipolar voltage map and the size as determined using cMRI. Mean (black line) ±2SD (blue line). C. Regression equation between the size of the non-transmural infarction as determined on a bipolar voltage map and the size as determined using cMRI. D. Bland-Altman plot of the size of the non-transmural infarction as determined on a bipolar voltage map and the size as determined using cMRI. Mean (black line) ±2SD (blue line).

Using SI = 51–75% and NOGA BipV 0.8–1.9 mV to define non-transmural infarction, the correlation between the two images was r = 0.555 (p<0.001). NOGA bipolar mapping resulted in a smaller area of non-transmural infarction, with a mean difference of 3.04±5.92% between the two images (Fig. 8).

Using SI = 31–60% and NOGA BipV 0.8–1.9 mV as cut-offs for non-transmural infarction, the correlation was slightly higher (r = 0.657, p<0.001), with a mean difference between the two images of 3.23±4.56% of LV surface.

### Overlap ratio

The overlap ratio was 81.6±11.1% and 70.6±18.5% for delineation of the transmural and non-transmural infarction size on the cMRI and NOGA images, respectively. None of the images showed an overlapping accuracy below 60% regarding transmural infarction. Regarding non-transmurality, two of the 60 animals (3.3%) showed an overlapping ratio between 50% and 60%, which was considered moderate agreement between the two imaging methods.

The overlap ratio of the combined transmural and non-transmural infarction was 70.2±12.2%.

## Discussion

Our results indicate that NOGA electroanatomical mapping is accurate enough to guide targeted intramyocardial therapy if both unipolar and bipolar maps are used. The results of NOGA were comparable to those of cMRI-LE, regarding the extent of the transmural and non-transmural scars and viable and reduced viability myocardial areas.

The main difference between the two 3D images is that different sampling methods are used to construct them. The NOGA 3D LV shape is constructed by point-to-point measurements, which is in contrast with the other 3D methods, such as cMRI-LE, which have a spatial resolution of 1.4 mm per voxel. For map construction, a 15-mm point-to-point distance was used by endocardial mapping, while an in-plane resolution of 1–2 mm with an 8-mm slice thickness was utilized for cMRI-LE analysis. Accordingly, the accuracy of the NOGA map depends mainly on the number of gathered and measured points and on the quality of the acquired points used to construct the 3D map. Difficulties in reaching some regions with the mapping catheter due to LV cavity structures such as the papillary muscles or the occurrence of ventricular arrhythmias due to touching vulnerable ischemic areas might lead to insufficient sampling, resulting in incomplete mapping. Nevertheless, unipolar voltage mapping overestimated the infarct size by just 2.82% of the entire LV endocardial surface, and bipolar mapping overestimated the transmural scar by just 6.82% of the LV. These estimates are acceptable for performing intramyocardial procedures safely and accurately. The overestimation of the infarct size and transmural scar as compared to cMRI-LE might result from the quantitative comparison of 2D NOGA polar maps and 3D cMRI-LE and 2D transmurality cMRI-LE images. According to our results, both the unipolar and bipolar voltage maps showed wider zones of interest than on cMRI, which may raise the risk of injecting reparative substances into normal myocardium. The intramyocardial injections are oriented into the non-transmural infarct or into areas with decreased viability. Different cut-offs are suggested for viability and transmurality thresholds in the literature; there are no universally agreed-upon definitions for these terms. Of course, the operators should try to make sure that the intramyocardial injections are made into the correct area, but an injection may be made into the “normal” myocardium if stronger cut-off criteria for non-normal territory are used. However, regenerative cells or other material delivered into the lane neighboring the non-transmural infarction have a good chance of being retained, being functional, and migrating or penetrating into the ischemic area, so a wider zone might result in more effective and safe treatment.

The sizes of the unipolar voltage scar and border zone differed from the sizes of transmural and non-transmural infarction as determined on the bipolar maps. Although the total size of the unhealthy areas on the unipolar and bipolar polar maps showed good correlation, the size of the infarction and border area determined on the unipolar maps was systematically larger than the size of the transmural and non-transmural regions on the bipolar maps. This is not surprising, because an infarct area contains heterogeneously viable myocytes that can be apoptotic or hibernating. Accordingly, the infarct core and the border area (i.e. the extent of infarction) do not necessarily overlap the transmural and non-transmural infarction (i.e. the infarction severity) because the infarct core may include transmural and non-transmural necrotic areas.

The unipolar voltage values represent a summation of the action potentials of the surrounding endocardial surface areas, while the bipolar voltage values represent the amplified difference between the tip of the unipolar electrode and the additional proximal electrode placed within the catheter. Accordingly, a non-transmural infarction with severe endocardial (but not epicardial) necrosis may appear to be an infarct core in the unipolar map. This might explain the differences between the unipolar and bipolar NOGA maps, and between the NOGA maps and other 3D images that measure infarct size. Nevertheless, using both (uni- and bipolar) maps of the NOGA images, information about both the extent and the severity of infarction are available real-time, on-table during a procedure, in contrast to off-line images such as cMRI-LE or myocardial single photon emission computed tomography.

Additionally, to set the correct and right cut-off values of the NOGA maps is of great importance. Several authors used several different cut-off values for definition of viability using the unipolar voltage maps, while the usage of bipolar maps is less common, probably due to the small range of not normal values (0–1.9 mV of interest) suggesting less precise differentiations. We have measured the transmural and non-transmural scars on the cMRI-LE image using two different SI cut-offs, because a SI between 25% and 50% suggests a false positive non-transmural infarction. When the cMRI transmurality threshold is lowered to 60% from 75% and to 31–60% from 51–75%, the size of the transmural and non-transmural infarct areas increase, and the correlations are slightly lower and higher, respectively. The SI of 30% and 60% for cut-off of non-transmural and transmural scars gave a better correlation with slight overestimation of the non-transmural scar by 3.23% of the LV. Since the endocardial injections should target non-transmural infarct areas, higher correlation between the two images in that region is desirable.

### Comparison of our results with literature data

The need to localize the arrhythmogenic focus for ablation during catheter-based electrophysiological procedure prompted several research groups to develop hybrid software that analyzes the 3D images of both NOGA maps and cMRI-LE. Fusion of cMRI with electroanatomical mapping of the LV proved to be successful with a registration error of 3.8±0.6 mm [22], with visual mismatches between the NOGA scars and LE in cMRI, particularly in patients with inferior infarction [22]. To enhance the accuracy of the electroanatomical mapping relative to cMRI-LE for the determination of myocardial scars with a presumed arrhythmogenic focus, different cut-off values were tested: 1.5 mV for non-transmural scars and 0.5 mV for dense scars [22]; 1.0 mV [28], 1.3 mV [23], 1.54 mV [24], 1.55 mV [26], and 1.9 mV [27] for bipolar maps; and 5.8 mV [23], 6.52 mV [24], 6.9 mV [18], 6.78 mV [26], 6.7 mV [27], and 5.1 mV [28] for unipolar maps. This reflects the uncertainty of finding the arrhythmogenic focus with electroanatomical mapping alone and emphasizes the visual mismatch between the two images in certain cases. In contrast with these studies, the sample size in our animal study was much larger (n = 60), but we did not use fusion software. Instead, we compared the 2D maps of the NOGA images with the 3D LE and 2D transmurality map of the cMRI. Nevertheless, we did not search for a single focus but rather for an area with reduced viability.

## Limitations

We did not use local linear shortening maps of the NOGA for delineating of the hibernating myocardium characterized by preserved viability and decreased segmental wall motion [12], because of the lower accuracy of the local linear shortening map and higher discordance with cMRI [9] regarding the segmental wall motion abnormalities. We overlaid the two images in order to determine the overlapping accuracies, while other research groups used 3D fusion software. Accordingly, our method has several shortcomings in the technique used. The study reports comparisons of 2D transmurality cMRIs and NOGA bipolar maps as well as comparisons of the 2D projection of the NOGA-derived infarct size and the 3D cMRI-LE-derived infarct size. However, both imaging modalities for quantitatively determining infarct size use standardized image processing functions of the software and are widely used.

We are aware that there are ongoing efforts to fuse 3D NOGA endocardial maps and cardiac MRIs to determine the similarities of infarct location and extent. Although numerous percutaneous intramyocardial therapy studies are currently underway with the aim of determining the optimal injection location, currently there is no commercially available 3D MRI-NOGA fusion software. The comparisons of cMRI and NOGA polar maps published previously lack some of the refinements we used, such as comparison of the transmurality map with the bipolar map and calculation of the overlapping ratio.

Unfortunately, we could not perform 3D volumetric co-registration of the compared modalities, as currently we do not have an image-processing tool with which to conduct such a comparison. The spatial correspondence was ensured by (1) similar selection of the basal and apical slice positions and (2) similar orientation of the bull's eye maps. The definition of sector orientation was based on manual selection of the right ventricular insertion point (sector 0) on the most basal MRI slice of the left ventricular myocardium. A real value of the fusion of the off-line cMRI-LE and on-table NOGA endocardial mapping would be the real-time display of the actual catheter position and location of the intramyocardial therapy on the reconstructed hybrid (cMRI and NOGA) image; which software is currently not available.

We have included the NOGA-derived volumetric measurements. The similarities between the NOGA-derived and cMRI-derived end-diastolic and end-systolic volume and calculated stroke volume and ejection fraction (Tables 2 and 3) ensure the acquisition of the entire LV map of the NOGA procedure and also ensure that the two images are comparable.

The use of 2SD method of cMRI-LE might overestimate the infarct size, as compared with the 5SD or “full width at half maximum” (FWHM) method. In our experience, it has not been the case that the 5SD method better reflects the infarct size compared to the 2SD approach, and there are data in the literature that show a higher correlation coefficient for the 2SD approach than the 5SD approach [33]. The FWHM method might correlate better with post-mortem measurements, but this approach has not yet been implemented into the software we used for our data analysis. Based on the different sampling modes and standardized segmentation of both MRI (17 segments divided into 6 basal, 6 mid and 5 apical segments) and NOGA (9 segments divided into 4 basal, 4 mid and 1 apex segments), direct comparison of segmental mean values to search for a mathematical cut-off for unipolar and bipolar values based on MRI cut-off values would require extensive digital processing and adaptation of both images. However, we correlated the unipolar and bipolar voltage values and calculated different bipolar voltage cut-offs using ROC analysis. Additionally, we have determined the optimal bipolar cut-offs using iterative thresholding and calculated the transmural and non-transmural infarct areas using 3 cut-offs and compared these results with those of the cMRI-LE data. However, we did not search unipolar voltage threshold values based on cMRI-LE images because voltage maps correspond to myocardial viability and cMRI is not the first-choice imaging method for viability assessment. Extensive research has been performed to establish cut-offs for viable, non-viable, and hibernating myocardium using imaging technologies such as myocardial scintigraphy and positron emission tomography [9]. We used these literature-based cut-off values to calculate infarct size.

## Conclusions

Our results demonstrate the usefulness of unipolar and bipolar maps generated using real-time electroanatomical mapping for targeted intramyocardial regenerative therapy. NOGA mapping showed good concordance with the off-line gold standard, cMRI-LE imaging. NOGA mapping may be useful in patients with contraindications for cMRI who require targeted intramyocardial regenerative therapy.
